# Acute Unilateral Vestibular Failure Does Not Cause Spatial Hemineglect

**DOI:** 10.1371/journal.pone.0135147

**Published:** 2015-08-06

**Authors:** Julian Conrad, Maximilian Habs, Thomas Brandt, Marianne Dieterich

**Affiliations:** 1 Department of Neurology, Ludwig-Maximilians-University Munich, Munich, Germany; 2 German Center for Vertigo and Balance Disorders—IFB^LMU^ (DSGZ), Munich, Germany; 3 Clinical Neuroscience, Ludwig-Maximilians-University Munich, Munich, Germany; 4 Munich Cluster for Systems Neurology (SyNergy), Ludwig-Maximilians-University Munich, Munich, Germany; Birkbeck, University of London, UNITED KINGDOM

## Abstract

**Objectives:**

Visuo-spatial neglect and vestibular disorders have common clinical findings and involve the same cortical areas. We questioned (1) whether visuo-spatial hemineglect is not only a disorder of spatial attention but may also reflect a disorder of higher cortical vestibular function and (2) whether a vestibular tone imbalance due to an acute peripheral dysfunction can also cause symptoms of neglect or extinction. Therefore, patients with an acute unilateral peripheral vestibular failure (VF) were tested for symptoms of hemineglect.

**Methods:**

Twenty-eight patients with acute VF were assessed for signs of vestibular deficits and spatial neglect using clinical measures and various common standardized paper-pencil tests. Neglect severity was evaluated further with the Center of Cancellation method. Pathological neglect test scores were correlated with the degree of vestibular dysfunction determined by the subjective visual vertical and caloric testing.

**Results:**

Three patients showed isolated pathological scores in one or the other neglect test, either ipsilesionally or contralesionally to the VF. None of the patients fulfilled the diagnostic criteria of spatial hemineglect or extinction.

**Conclusions:**

A vestibular tone imbalance due to unilateral failure of the vestibular endorgan does not cause spatial hemineglect, but evidence indicates it causes mild attentional deficits in both visual hemifields.

## Introduction

Neglect is a heterogeneous disorder of spatial attention. It is characterized by reduced awareness of multisensory stimuli in the hemifield contralateral to a frontal or temporo-parietal lesion, mainly in the right hemisphere. In previous decades neglect subtypes were differentiated on the behavioural level. Neglect can involve several aspects of the representation of space: egocentric or allocentric (object centered), (peri-) personal and far space, visuospatial receptive functions, as well as motor-intentional representation of space. Even more abstract concepts of spatial representation and organization such as the location of objects in an imagined space (representational neglect) or the location of numbers on a line (neglect in the number space) have been discussed. [1]

The cortical areas involved patients that show neglect behavior are the inferior parietal lobule at the temporo-parietal junction,[2] the posterior intraparietal sulcus, and the middle frontal gyrus.[3] The superior temporal gyrus and insula are also involved.[4, 5] Recent evidence indicates that neglect results when the aforementioned cortical regions are disconnected by lesions of long-range white matter pathways joining the frontal and parietal lobes.[6] Some of these same regions that are lesioned in patients with spatial neglect form part of the cortical multisensory vestibular network.[5, 7–9]

Not only is vestibular integrity important for maintaining visuospatial maps for spatial orientation,[10–12] vestibular caloric stimulation (with cold water of the contralesional ear) significantly improves spatial functioning, a sign that the vestibular system plays a role in neglect.[13–15] The modulation of hemineglect by vestibular stimulation, especially when combined with neck muscle vibration, seems to provide the important sensory signals needed to create a frame of reference for space perception based on the coordinates of eye and head position in space.[16] This consequently raised the question of whether neglect could represent a cortical disorder of higher vestibular function.[17] It has even been speculated that neglect is not only a cortical disorder but may occur with peripheral and central vestibular pathway lesions.[18] In fact, a recent study reported that patients with vestibular neuritis can have pathological scores in paper-pencil neglect testing.[19] The authors concluded that these patients showed mild spatial neglect either to the ipsilateral or contralateral side. However, the reported test results, in particular the attentional deficits ipsilateral or contralateral to the side of the affected ear, are incompatible with the pathophysiological concept of spatial hemineglect.

We therefore tested patients with an acute unilateral vestibular failure (VF) due to vestibular neuritis or Menière’s disease with various neglect tests that were correlated with the severity of the vestibular tone imbalance indicated by tilts of the subjective visual vertical for otolith dysfunction and hyporesponsiveness in caloric stimulation for semicircular canal paresis.

## Patients and Methods

Twenty-nine patients with acute unilateral VF who were admitted to the Department of Neurology, Munich University hospital, were studied prospectively. The diagnosis was based on the clinical findings of horizontal rotatory spontaneous nystagmus with the fast phase beating to the contralateral ear and a deficient vestibulo-ocular reflex (VOR) in the head-impulse test. Patients with clinical signs of central involvement (skew deviation, bilateral gaze-evoked nystagmus, disturbed fixation-suppression of the VOR) were not included. One of these patients was excluded due to prior sensory deficits (polyneuropathy, bilateral vestibulopathy). The remaining 28 patients were included in the study. Clinical testing was carried out by an experienced neuro-otological expert. Patients underwent orthoptic testing, which included measurements of the subjective visual vertical (SVV) in 26 patients. The degree of canal paresis was analyzed in 23 patients using Jongkees’s formula for the nystagmus induced by caloric irrigation.[20] Orthoptic testing could not be performed in two patients, and caloric testing, not in five patients due to severe vertigo and vomiting.

All patients showed clinical signs of VF at the time of testing. The mean time from symptom onset to the completion of the neglect tests was 1.8 days (SD 1.7, max. 6 days). None of the patients had aphasia, hemianopia, or clinically significant cognitive deficits. Patient characteristics are given in Table 1.

**Table 1:** Patient demographic and clinical data.

	**No.**	
Number of patients included in analysis	28	
Age (in years, SD)		59.2 (15.0)
Educational level (mean years of school, SD)		11.0 (2.0)
	**No.**	**%**
**Gender**		
Female	15	53.6
Male	13	46.4
**Handedness**		
Left	2	7.1
Right	26	92.9
**Vestibular failure (VF) side**		
Left	13	46.4
Right	15	53.6
**VF etiology**		
Vestibular neuritis	26	92.9
Menière’s disease	2	7.1
**Orthoptic testing**		
Yes	26	92.9
No	2	7.1
**Caloric Testing**		
Yes	23	82.1
No	5	17.9
**MRI**		
Yes	20	71.4
No	8	28.6
**Neglect Tests completed (number of patients)**	**No.**	**%**
Albert’s Test	28	100
Bells Test	26	92.9
Line bisection Test (LBT)	28	100
Figure copying	18	64.3
**Time from symptom onset to test completion**	**days (d)**	**SD (d)**
Orthoptic examination	1.9	1.8
Caloric irrigation	2.3	2.3
Neglect tests	1.8	1.7

For the measurement of SVV a mean of more than 2.5° of the seven measurements of the static SVV determined binocularly was considered pathological.[21]

Spatial neglect was diagnosed on the basis of spontaneous behavior, for example, if patients showed a constant deviation of head and eyes to one side or failed to properly respond and orient themselves to stimuli presented on the contralateral side when approached or addressed verbally. Visual extinction was tested using a confrontation technique. Patients had to determine whether the examiner’s index finger moved in the left, right, or both hemifields, in either the upper or lower quadrants. To determine somatosensory extinction, patients were lightly touched on their forearm, either on the left, right, or both sides simultaneously. Auditory stimuli (snipping sounds) were presented on either side or simultaneously directly in front of the ears. All stimulus conditions were repeated in random order three times for each presentation (left, right, both). Patients were asked to identify the side of the stimulus. Extinction was diagnosed if patients failed to detect bilateral presentation of the stimuli in any of the runs.

Visuo-spatial functions were further tested by a standardized paper-pencil line bisection task,[22] and two cancellation tasks, the modified Albert’s test,[23, 24] and the Bells test.[25] In addition, a figure-copying task was performed by 18 patients.[26]

In the line bisection task three solid lines have to be bisected on a standard sheet of paper. A score from 1–3 is given for the distance from the true center of each line (a mark farther from the midline leads to a lower score; the highest score is 9 and reflects normal peripersonal spatial function). A score < 7 was considered pathological.[22] The cancellation tasks were evaluated as follows: one or more omissions on one side in Albert’s test,[24] and six or more omissions on one side in the Bells test were considered pathological.[25] The figure-copying task was evaluated visually. The results of the cancellation tasks were further analyzed with the Center of Cancellation (CoC) method established by Binder and co-workers, which was recently validated by Rorden and Karnath.[27, 28] The continuous scoring of this method is sensitive to both the number as well as the location of these omissions. Scores approaching positive or negative one indicate left- or right-sided neglect, respectively. The cut-off level for pathological test scores was set at 0.081; this was also proposed for right hemisphere stroke.[28]

The Bells test could not be carried out in two patients who were unable to distinguish the bells from the distractors on the test sheet due to impaired vision.

### Statistics

To relate omissions on the Bells test to vestibular dysfunction a Kendall’s tau correlation was carried out. Normal distribution was tested using the Shapiro Wilk test, equality of variances was determined using Levene’s test. Differences between the groups with any pathological neglect test score and those without were tested using t-test statistics for independent variables. The values of the deviation of SVV were log-transformed to achieve normal distribution. Significance level was set to p < 0.05.

### Standard protocol approvals, registrations and patient consent

The study was performed in accordance with the 1964 Declaration of Helsinki and was approved by the institutional review board of the University of Munich. The aim of the study and their inclusion were discussed with the patients and they consented verbally to participate in the study. All tests used in the study were part of the routine workup to determine the cause of vestibular failure (caloric testing, orthoptic testing, MRI to distinguish peripheral from central vestibular dysfunction) and to explore the need for specific rehabilitative measures if the patients showed neglect (clinical and paper-pencil neglect tests). I. e. these routine clinical measurements had to be carried out regardless of the decision to participate in the study. Patients were carefully informed about the study and consent was documented on a separate consent sheet before obtaining the patients demographic and clinical data. Additional written consent was obtained from healthy participants and patients who participated in the fMRI experiment. This consent procedure was approved by the institutional review board.

## Results

On the basis of the clinical criteria none of the patients showed spatial neglect or tactile, visual, or auditory extinction. In the paper-pencil testing one patient fulfilled the criteria for contralesional spatial hemineglect (one omission) in Albert’s test. Another patient fulfilled the criteria for contralesional spatial hemineglect in the Bells test. When measuring neglect severity in the Bells test, the mean CoC value was +/- 0.023 (SD 0.027) in 26 patients; seven patients had a CoC score of 0. One patient showed a pathological orientation bias in the Bells test (score 0.088) to the ipsilesional side (see also S1 Table).

The mean score was 8.65 in the line-bisection task (mean distance from the center +/- 0.31 cm, max: 2.3 cm). One patient had a pathological score of 4 but showed a leftward and rightward deviation within the same test. There was no inter-test predictability of a pathological result.

A pathological tilt of SVV was found in 23 of 26 patients; the mean SVV tilt (of 7 measurements binocularly) was 8.3° (SD 6.7°). The percentage of canal paresis was 51.3% (SD 26.8%); the mean slow phase velocity (SPV) of spontaneous nystagmus was 6.3°/s. Minor central ocular motor pathology was observed in five patients (disturbed vertical smooth pursuit only).

There was no correlation between the severity of the vestibular tone imbalance and the number of omissions in the Bells test (for deviation of SVV: Kendall’s Tau-b = 0.12, p = 0.43, normal distribution of omissions in the Bells test p = 0.005; for canal paresis: Kendall’s Tau-b = -0.2, p = 0.17, for slow phase velocity of spontaneous nystagmus: Kendall’s Tau-b = -0.19, p = 0.23). The mean deviation of the SVV was 11.27 (SD 4.9°) in patients with any pathological test result compared to 7.9° (SD 6.8°) in patients without such findings. Mean reduction in caloric excitability was 55.2% (SD 5.4%) and 50.2% (SD 28.5%) in the respective groups (see also S2 Table). There was no significant difference in the means of the tree measurements (t-test for independent variables: p = 0.27 for deviation of SVV, for canal paresis p = 0.84, for slow phase velocity of SPN p = 0.56). It has to be noted that the group with pathologic scores consisted of only three (two for canal paresis statistics) patients. Therefore the results should be interpreted with caution.

## Discussion

Standard neglect testing for peripersonal neglect in our series of 28 patients with acute VF did not reveal signs of a typical spatial hemineglect. The few pathological test results of three of the 28 patients are best attributed to attentional deficits, which could appear ipsilaterally or contralaterally to the lesioned side. Therefore, we are unable to confirm the conclusions drawn by others, which were based on similar heterogeneous deficits.[19] Our results must be interpreted with caution, since the group that showed pathological test results is small. It is necessary to consider that other factors such as age or gender might have confounded the results. However, several studies have proven that the cut-off values we also used in our study reliably distinguish patients with neglect from those without regardless of age, gender, or handedness.[22–28] Moreover, several authors have examined the performance in line bisection tasks in healthy volunteers.[29] They were able to demonstrate a leftward deviation in line bisection in healthy volunteers, a finding termed “pseudoneglect” by Bowers and Heilman.[30] Age-related changes (e.g., a more rightward bias) and also gender differences are reported in the literature. These findings are much less pronounced in normal than in neglect patients and clearly differ from our findings. Our results did not show corresponding pathological test scores in different tests or analysis methods and no clear predominance of omissions on the excentric part of one side of the test sheet. Thus, we favor the view that the pathological findings represent an attentional deficit due to the acute vestibular failure rather than just age-related changes.

Our results pose two major questions: (1) What are the different functional consequences of a peripheral vestibular tone imbalance compared to a cortical imbalance for multisensory spatial orientation? (2) Are the methods used sufficient to exclude subtle neglect symptoms, e.g. possibly restricted to the periphery of the visual field, far space or mental representation of space? In the presence of temporo-parietal lesions including the multisensory vestibular network, the processing of sensory stimuli in the contralateral visual hemifield is impaired, i.e., right hemispheric lesions impair awareness of visual stimuli in the left hemifield. In contrast, if an acute unilateral peripheral vestibular failure occurs, both hemispheres perceive the vestibular tone imbalance similarly because the ascending pathways transmit signals via uncrossed ipsilateral as well as crossed contralateral pathways to both temporo-parietal cortices. Therefore, the vestibular signals from both sides are processed in each hemisphere and do not lead to a functionally relevant cortical asymmetry of “vestibular” spatial orientation. [31, 32] This may impair attention in both hemifields, but obviously it does not cause signs and symptoms of a typical spatial hemineglect. Indeed, the pathology of the peripheral vestibular endorgan can cause a range of cognitive deficits, not only spatial but also non-spatial such as a deficiency in object recognition memory.[33, 34] The attentional deficits can be attributed to the highly distressing vertigo and nausea in the acute stage. Further, significant deficits in spatial memory and navigation have been demonstrated in rodents as well as in patients with chronic *bilateral* vestibular deafferentiation.[35, 36]

It might be worthwhile to test the deviation of the subjective straight-ahead in these patients rather than the tilts of SVV and to look with more specific methods for attentional deficits especially in the periphery of the visual field contralateral to the side of the lesion. The latter appears promising, since vestibular stimulation like caloric irrigation was able to shift the meridian of the hemineglect in the direction of the slow phase of caloric nystagmus.[16] We also focused on the occurrence of personal and peripersonal neglect in patients with acute vestibular failure. We cannot exclude the possibility, however, that subtle spatial deficits involving other subtypes of neglect and more complex tasks might also rely on vestibular cues such as spatial navigation, the mental representation of space (representational neglect), or spatial orientation in far space.

## Supporting Information

S1 Table Results neglect tests. (DOCX)

S2 Table Results: Subjective visual vertical (SVV), caloric irrigation and mean slow phase velocity of spontaneous nystagmus (SPN). (DOCX)
