# Supplementary material for: Acute Unilateral Vestibular Failure Does Not Cause Spatial Hemineglect
Source: PLoS One. 2015 Aug 6;10(8):e0135147. doi: 10.1371/journal.pone.0135147 (PMC4527734; doi:10.1371/journal.pone.0135147)
Supplement: S1 Table — (DOCX) [file pone.0135147.s001.docx]

| **S1 Table Results neglect tests** | | | | | |
| --- | --- | --- | --- | --- | --- |
|  | | **Unremarkable** | | **Pathological** | |
|  | | No (%) | | No (%) | |
| Albert’s test  Bells test (n=26)  LBT | | 27 (96.4)  25 (96.2)  27 (96.4) | | 1 (3.6)  1 (3.8)  1 (3.6) | |
|  | | **Mean (SD)** | | **Mean (SD)** | |
| Center of cancellation (CoC)  Bells test | | 0.02 (+/-0.027) | | 0.088 (only one value) | |
| Mean Score LBT | | 8.86 (+/- 0.36) | | 4 (only one value) | |
|  | |  | |  | |
|  |  | |  | |  |
|  |  | |  | |  |
