# Supplementary material for: Acute Unilateral Vestibular Failure Does Not Cause Spatial Hemineglect
Source: PLoS One. 2015 Aug 6;10(8):e0135147. doi: 10.1371/journal.pone.0135147 (PMC4527734; doi:10.1371/journal.pone.0135147)
Supplement: S2 Table — (DOCX) [file pone.0135147.s002.docx]

| **S 2 Table Results: Subjective visual vertical (SVV), caloric irrigation and mean slow phase velocity of spontaneous nystagmus (SPN)** | | | |
| --- | --- | --- | --- |
|  | **Total** | **Neglect score unremarkable** | **Neglect score pathological** |
| **Mean SVV (SD) in °** | 8.3° (6.7°) | 7.9° (6.8°) | 11.27° (4.9°) |
| **Canal paresis (SD) in %** | 51.3% (26.8) | 55.2% (5.4%) | 50.2% (28.5%) |
| **Mean slow phase SPN (SD) in °/s** | 6.3°/s (4.3 °/s) | 6.4 °/s (4.3 °/s) | 4.5 °/s (6 °/s) |
